# Evaluation of *Ricinus communis* L. for the Phytoremediation of Polluted Soil with Organochlorine Pesticides

**DOI:** 10.1155/2015/549863

**Published:** 2015-08-02

**Authors:** Sandra Regina Rissato, Mário Sergio Galhiane, João Roberto Fernandes, Marli Gerenutti, Homero Marques Gomes, Renata Ribeiro, Marcos Vinícius de Almeida

**Affiliations:** ^1^Department of Chemistry, Paulista State University (UNESP), CP 473, 17033-360 Bauru, SP, Brazil; ^2^Department of Physics, Federal University of São Carlos (UFSCar), 13565-905 São Carlos, SP, Brazil; ^3^Laboratory for the Toxicological Research (Lapetox), University of Sorocaba (UNISO), 18023-000 Sorocaba, SP, Brazil; ^4^Department of Physics, Chemistry and Biology, Paulista State University (UNESP), 19060-900 Presidente Prudente, SP, Brazil

## Abstract

Phytoremediation is an attractive alternative to conventional treatments of soil due to advantages such as low cost, large application areas, and the possibility of in situ treatment. This study presents the assessment of phytoremediation processes conducted under controlled experimental conditions to evaluate the ability of *Ricinus communis* L., tropical plant species, to promote the degradation of 15 persistent organic pollutants (POPs), in a 66-day period. The contaminants tested were hexachlorocyclohexane (HCH), DDT, heptachlor, aldrin, and others. Measurements made in rhizosphere soil indicate that the roots of the studied species reduce the concentration of pesticides. Results obtained during this study indicated that the higher the hydrophobicity of the organic compound and its molecular interaction with soil or root matrix the greater its tendency to concentrate in root tissues and the research showed the following trend: HCHs < diclofop-methyl < chlorpyrifos < methoxychlor < heptachlor epoxide < endrin < o,p′-DDE < heptachlor < dieldrin < aldrin < o,p′-DDT < p,p′-DDT by increasing order of log *K*_ow_ values. The experimental results confirm the importance of vegetation in removing pollutants, obtaining remediation from 25% to 70%, and demonstrated that *Ricinus communis* L. can be used for the phytoremediation of such compounds.

## 1. Introduction

Persistent organic pollutants (POPs) are relatively inert, and their high stability is related to aromatic ring, carbon-chlorine bond, and other chemical arrangements. These compounds are widely studied due to their high toxicity, low biodegradability, and biosolubility in fat tissue [1]. Some of these compounds may persist for 15 to 20 years in soil and part of these are entrained by rain (leaching) into water courses, which also receives these compounds by industrial effluents, sewage, sediment, and air and by direct contamination during use [2].

Compounds such as organochlorines accumulate along the food chain, and much remains in the environment and can contaminate water and food making them unsuitable for consumption [3]. Therefore, they represent the most persistent organic pollutants (POPs) prioritized by United Nations Environmental Programme (UNEP) and banned or restricted by the Stockholm Convention in May 2001 [4].

Organochlorine pesticides such as dichlorodiphenoxytrichloroethane (DDT) and its metabolite p,p′-dichlorodiphenoxydichloroethylene (p,p′-DDE) and hexachlorocyclohexane (HCH) are more successful in the chemical control of pests and have been used in agriculture and public health activities (eradication of malaria and other vectors) in the world in the past decades, but its use still remains in many developing countries. In Brazil, organochlorine pesticides were used to control pests and increase food production during the 70s [5]. Among them, pesticides as DDT, HCH, heptachlor, aldrin, dieldrin, and endrin were the most extensively used. Although its use has been discontinued in Brazil since 1985, the effect of half-life leads to its persistence, which has generated considerable amounts of these compounds in the environment [5, 6]. Currently, the use of DDT is still allowed in public health programs, such as the fight in etiologic vectors (malaria and leishmaniasis) as well as in emergency. Historically, South America is considered the continent with the greatest use of DDT, lindane, and toxaphene [5].

Scientific methods must be undertaken to create innovative technologies for the cleanup of contaminated soil to minimize this impact. Physical, chemical, and biological methods have often been used to remediate contaminated sites [7].

Today, however, phytoremediation is proposed as a cost-effective method for the removal or treatment of many classes of contaminants such as petroleum hydrocarbons, chlorinated solvents, pesticides, metals, radionuclides, explosives, and excess nutrients [8–10]. In addition, this technique stands out for its efficiency in immobilization of pollutants in their tissues and due to its financial return that can be achieved by the sale or sales of biomass generated during decontamination of given area [11].

The mechanism that governs this process depends primarily on the physicochemical parameters of organochlorine pollutants (OCPs), such as their hydrophobicity (lipophilicity), solubility, polarity, or molecular weight, and on parameters involved in the metabolism of the plant or microorganism [5]. It applies primarily to hydrophilic or moderately hydrophobic compounds (log *K*_ow_: 0.5–3.0) but does not apply to highly hydrophobic compounds as POPs. Hydrophobic chemicals such as POPs have octanol-water partition coefficient (log *K*_ow_) values ranging from 3.0 to 8.3 (Table 1) [12]. Sicbaldi et al. [13] studied the potential of the soybean plants testing hydrophobic compounds with log *K*_ow_ values between 2 and 3. The compounds are translocated most efficiently within the vegetation, and the translocation efficiency decreased for compounds with higher log *K*_ow_ values [14].

Highly hydrophobic pesticides easily permeate plasma membranes but do not partition well into the xylem sap due to their affinity for lipidic sites in the cell [13].

In recent years, bioremediation procedures have focused on phytoextraction and phytoremediation to clean up soils contaminated with organic pollutants as polycyclic aromatic hydrocarbons (PAHs) and POPs [15, 16]. Recent studies have demonstrated the importance of understanding the relationship between bacterial activity generated by the plant and remediation process of contaminated soil with DDTs and HCHs [17].

In addition, other researches showed the uptake of polychlorinated biphenyls (PCBs) and p,p′-DDE by species* Cucurbita pepo* [18, 19].* Avena sativa* L.,* Chenopodium *spp.,* Solanum nigrum* L.,* Cytisus striatus* (Hill) Roth, and* Vicia sativa* L. were recently investigated to evaluate their use in the phytoremediation of soil contaminated with hexachlorocyclohexane isomers. These plants proved particularly potent in accumulating the *β*-HCH isomer in their tissues [20].

Castor bean (*Ricinus communis* L.), a dicotyledonous plant belonging to the family Euphorbiaceae, includes a large number of native species in the tropical region from Ethiopia, Africa [21]. It is an oil seed crop of importance in Brazil and worldwide [22].

Its oil is a raw material which has hundreds of versatile applications in chemical industry which can make several reactions giving rise to various products, ranging from the manufacture of lubricants and grease, paints, varnishes, foams, and plastic materials for different purposes to the production of cosmetics production food, pharmaceutical, and products for the automotive industry [23].

Beyond wide application in the chemical industry, the castor bean is a resistant plant that also has economic advantages when used in the remediation of soil contaminated with heavy metals [23, 24].

This work presents the results from a greenhouse-scale study of phytoremediation of POPs-contaminated soil using castor bean (*Ricinus communis* L.). Specifically, this study examines the potential of* Ricinus communis* L. (castor bean) for phytoremediation of 15-POPs-contaminated soil at two concentrations. The application of this process to polluted soil will promotemaintenance/replacement of nitrifying bacteria, restoring its natural and organic status, providing security to human and animal health, and enabling the use of these fields for economic and social activities.

## 2. Materials and Methods

### 2.1. Reagents

All the chemicals and solvents were of a particular grade for pesticide residue analysis and purchased from Mallinckrodt Baker (Phillipsburg, NJ, USA). Purified water was obtained from a Milli-Q water purification system (Millipore, Bedford, MA, USA). Pesticide standards were obtained from Chem Service Inc., West Chester, PA.

Stock solutions of pesticides (approximately 500 *μ*g L^−1^) of mixed standards were prepared by dissolving about 0.050 g of the pesticides in 100 mL of toluene/n-hexane (1 : 1, v/v) and storing the mixtures in a freezer at −18°C in glass bottles with PTFE-faced screw caps. Pesticide working solutions were prepared by dilution in toluene/n-hexane (1 : 1, v/v). Sodium sulfate was pesticide grade. Silica gel, grade 634, 100–200 mesh, was used for sample extract cleanup.

### 2.2. Soil Preparation and Experimental Design

Soil core samples were collected from an experimental field close to the city of Bauru, state of São Paulo, Brazil. The soil samples were taken from the upper horizon (0–20 cm), air-dried, and sifted through a 2-mm sieve. Uncontaminated soil (previously solvent extracted and then analysed) was then spiked with a mixture of highly pure organochlorine pesticides in n-hexane/toluene (1 : 1, v/v). After the n-hexane/toluene evaporation, the spiked soils (4000 g dry weight of soil per pot) were then packed into plastic pots (14 cm tall and 15 cm in diameter). They were lined with gravel and sand, with a 0.1-mm sieve at the bottom to aid drainage and avoid soil loss [25].

The pots were placed in a greenhouse and maintained for seven days at field moisture and soils fertilized with 1 g of NPK fertilizer mixture (1 g kg^−1^ of soil) containing a ratio of N : P_2_O_5_ : K_2_O = 1.00 : 0.35 : 0.80 before receiving plants [26]. The plant employed in this experiment was castor bean (*Ricinus communis *L.) selected to reflect typical species found in the region and to cover a broad range of physiology and root morphology.

The experiments were identified as

 P_0_—soil + POPs;
 P_1_—soil + POPs + castor bean;
 P_2_—soil + castor bean.

The treatments were divided into the following groups: (a) unplanted pots with spiked soil, (b) planted pots in spiked soil, and (c) planted pots with unspiked soil (control). The seeding date was considered day 0 (zero). Pots containing planted and unplanted soil were placed in a greenhouse and temperature was kept at 25°C during the 16-h day and 19°C during the 8-h night.

Each test was conducted in triplicate, and the pots were placed randomly in the greenhouse. The plants were exposed to 2 different concentrations of organochlorine pesticides during the experiment: *T*_1_ = 1.0 *μ*g g^−1^ and *T*_2_ = 2.0 *μ*g g^−1^. At the end of the experiment (66 days), all the plant parts (leaf, stem, and root) were harvested, rinsed with tap water and distilled water, and separated into aerial (leaf and stem) and root components. Both shoots and roots were freeze-dried for maximum 7 days before analysis.

### 2.3. Analysis of Organochlorine Pesticides

#### 2.3.1. Soil

Five grams of dried and homogenized soil samples was extracted for 3 h in a Soxhlet extractor with 130 mL of pesticide grade n-hexane : acetone 1 : 1 v/v. After extraction, the samples were concentrated to a volume of about 5 mL using a rotary vacuum evaporator at 45°C. A cleanup column containing 6 g of silica gel topped with 2 cm of anhydrous sodium sulfate was washed with 2 × 15 mL hexane. The sample extracts were transferred to the column and eluted with 130 mL of hexane and 15 mL of dichloromethane [27]. The fractions were collected as a single fraction and concentrated to 5 mL in a rotary vacuum evaporator at 45°C and then further concentrated to 1 mL under a gentle stream of purified nitrogen gas. The extracts were stored in sealed bottles at −20°C prior to analysis. The recovery efficiency was evaluated by obtaining spiked soil samples (0.1 *µ*g g^−1^ for each compound; Table 2).

#### 2.3.2. Plants

About 30 mL of acetone : dichloromethane (3 : 1 v/v) suspension was added to 10 g of dried and powdered plant parts. After 20 min of maceration at room temperature, the samples were homogenized in a vortex mixer with 3 g of anhydrous Na_2_SO_4_ for another 5 minutes. The extraction process was followed by cleanup by solid phase extraction with Florisil. Glass columns (30 cm × 1.5 cm i.d.) were packed at the bottom with a glass/cotton wool plug and 5 g of Florisil (Sigma-Aldrich, 60–100 mesh size) with a top layer of 2 g of anhydrous Na_2_SO_4_. Samples were eluted with 30 mL of the same solvent mixture (acetone : dichloromethane 3 : 1 v/v), concentrated in a rotary evaporator and then reconstituted in 1 mL toluene and stored at 4°C for final analysis.

### 2.4. Analytical Procedure

#### 2.4.1. Gas Cromatograph/Electron Capture Detector (GC/ECD)

The Hewlett Packard HP 5890 Series II gas chromatograph was used, equipped with a ^63^Ni electron capture detector and a fused silica capillary column HP-608 (30 m × 0.25 mm i.d. and 0.25 *μ*m film thickness). The operating conditions were as follows: initial temperature of 45°C (1 min), increase to 150°C at 20°C min^−1^, holding for 5 min, then an increase to 280°C at 4°C min^−1^ for 20 min, injector temperature 250°C, carrier gas H_2_, column linear velocity (*μ* = 45 cm s^−1^), detector temperature 300°C, N_2_ makeup gas, splitless mode operation, 1 min purge-off time, and 1 *μ*L injection volume.

#### 2.4.2. Gas Chromatograph/Mass Spectrometer (GC/MS)

A confirmatory analysis was done on a Hewlett Packard HP 5890 Series II gas chromatograph with a HP 5972 mass selective ion detector (quadrupole) and a 5% phenyl-95% dimethyl polysiloxane DB-5 coated fused silica capillary column (30 m × 0.32 mm i.d., 0.25 *μ*m film thickness). The carrier gas was purified helium applied at flow rate of 1.5 mL min^−1^. Four microliters of sample was injected into the GC-MS in splitless mode, using an injection time of 1 min, with the injection temperature set at 280°C. The oven temperature for the OCP analysis was programmed from 70 to 140°C at a heating rate of 25°C min^−1^, 140 to 179°C at a rate of 2°C min^−1^, 179 to 210°C at 1°C min^−1^, and 210 to 300°C at 5°C min^−1^, where it was held for 10 min. The analysis was conducted in the Selective Ion Monitoring (SIM) mode and the mass spectrometer parameters were as follows: impact ionization voltage 70 eV, ion source temperature 230°C, transfer line 300°C, electron multiplier voltage 1200 V, solvent delay 2.9 min, electron scan rate 1.5 scan s^−1^, and scanned-mass range 40–600 m z^−1^.

## 3. Results and Discussion

### 3.1. Quality Assurance

Quantitative determination of all the samples was made by the external standard method, using peak area integration parameters. Linear calibration curves for all the pesticides were made at five calibration levels, from 0.005 to 0.5 *μ*g L^−1^, and all the standard calibration curves fell within the acceptable limits of the linearity criterion (data shown in Table 2).

The limit of detection (LOD) of individual target molecules was determined by the concentration of analysis in a sample that produced a peak with a signal-to-noise ratio (*S*/*N*) of 3.

The limit of quantification (LOQ) for all the target molecules was based on the GC/ECD performance and background noise levels under laboratory conditions. These parameters were determined by analyzing procedural blanks in the same bath, which were consistent (RSD < 30%). Therefore, the median empty value was used as a primary parameter for subtraction. The LOQ was calculated and established at three times the standard deviation, taking into account the blank level, and the results of each sample analysis showed about 95% certainty.

The method was validated using control plants and soil as blank samples spiked with 0.01 *μ*g g^−1^ and 10 × LOQ 0.1 *μ*g g^−1^. Plant tissues (root, stem, and leaf) and spiked soil samples (*n* = 5) were analyzed.

To gain a better understanding of the global cycle, an integrated study of the behavior of organic contaminants in the ground and plant is essential for the development of phytoremediation techniques that are applicable in contaminated regions throughout the world.

In the present work, the phytoremediation effect of the vegetable* Ricinus communis *L. was evaluated in spiked soil. HCH isomers (*α*, *β*, and *δ*), DDT and its metabolites (DDE and DDD), trans-chlordane, diclofop-methyl, aldrin, dieldrin, endrin, heptachlor, heptachlor epoxide, and methoxychlor were used as organochlorine pesticides. Table 2 lists the rates of recovery and precision of variations of organochlorine pesticides that were determined in spiked soils and plants.

### 3.2. Plant Uptake of Pesticides

The most important aspect of phytoremediation in large contaminated areas is the identification of the most suitable plants species that show successful uptake of target contaminants [26].* Ricinus communis* L. is an industrial crop with multiple nonfood uses and economic advantages including the remediation of heavy metal in contaminated soils [28].

**Figure 1:**
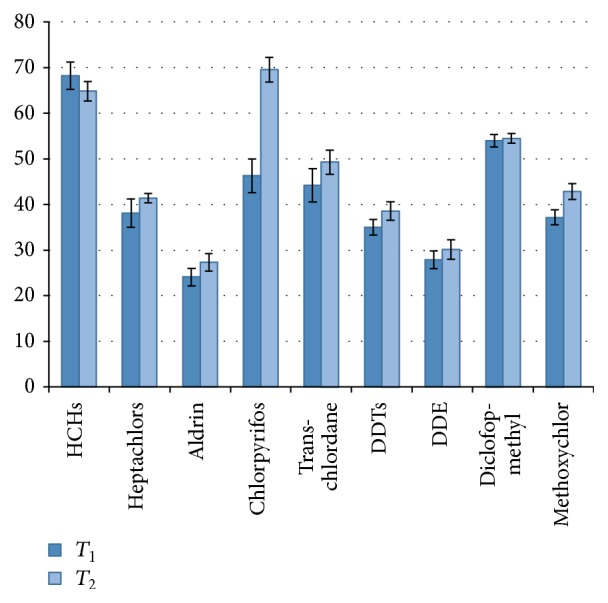
Distribution of pesticides obtained by experiment of phytoremediation using* Ricinus communis *L. in contaminated soil at 1.0 *μ*g g^−1^ (*T*_1_) and 2.0 *μ*g g^−1^ (*T*_2_) after 66 days.

In this work* Ricinus communis* L. showed high uptake of OCPs in contaminated soil. The results obtained at different concentrations of POPs (*T*_1_ and *T*_2_) were similar for most of the compounds studied (above 40%). However, for compounds aldrin and DDTs/DDE, remediation results observed were of 24.28–27.22% and 35.8–38.3%/28.33–30.29%, respectively. The results showed in Figure 1 were based on the found residues in the contaminated soil samples in relation to control samples (no plants) in *T*_1_ (1.0 *μ*g g^−1^) and *T*_2_ (2.0 *μ*g g^−1^) treatments after 66 days. The remediation in the presence of growing* Ricinus communis* L. showed restoration results of 24.28 to 68.33% in the *T*_1_ treatment and 27.33 to 69.01% in the *T*_2_ treatment (Figure 1).

Organic contaminants can accumulate in the roots essentially as a result of two processes: (i) uptake and translocation, for compounds with low hydrophobicity (log *K*_ow_ values between 0.5 and 3.5), and (ii) adsorption of root tissue [8].

The best results were found for the remediation of HCHs (65.07 to 68.33%), chlorpyrifos (46.34 to 69.01%), diclofop-methyl (53.66 to 54.98%), and trans-chlordane (44.17 to 49%). These compounds have log *K*_ow_ between 3.6 and 5.5 which may have contributed to the process of uptake and translocation. Studies have focused on the pollutant in the plant dynamics and transfer of pollutants and their bioconcentration in plant tissues physicochemically describe the importance of the octanol-water partition coefficient (*K*_ow_), the vapor pressure of organic pollutants, and ambient temperature, relating the mobility and solubility in water [29]. Ideally, such naturally nonpolar pollutants can be solubilized in water and transferred to plants. Matsumoto et al. proposed that root exudates eliminated by low molecular weight such as citric acid, other organic acids, and proteins can contribute to increased solubility of compounds such as POPs, and, furthermore, they mention a few plant families have this capacity [26].* Ricinus communis* L. has shown greater efficiency in the remediation of POPs compared to zucchini and pumpkin [30], alfalfa, or corn [31, 32]. These results are consistent with both Burkhard [33, 34] and Iannuzzi et al. [35] that concluded that the lipid content of the exposed organisms and the *K*_ow_ of the contaminant influence estimates of tissue concentrations more than other parameters.

However, many authors associate the presence of arbuscular mycorrhizal fungi buildup found in roots and that in turn increased the surface contact with soil with the efficiency of the remediation process.

**Figure 2:**
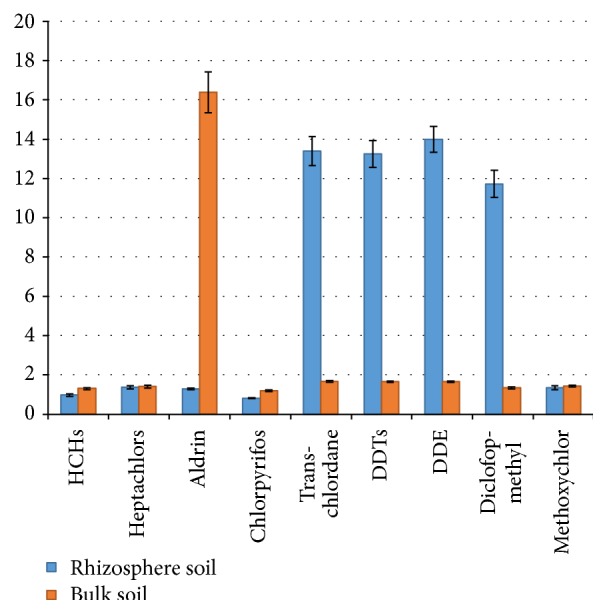
Distribution of pesticides in rhizosphere and bulk soil obtained by experiment of phytoremediation using* Ricinus communis *L. in spiked soil at 2.0 *μ*g g^−1^ (*T*_2_).

Figure 2 compares rhizosphere and bulk soil samples for the studied plant in contaminated soil at 2.0 *μ*g g^−1^ (*T*_2_). These results indicated that the rhizosphere soil presented a higher remediation for all the pesticides than the bulk soil. Interactions between the root system and its immediate surroundings (rhizosphere) may affect the behavior of these contaminants, modifying the system's physicochemical and microbiological properties and thus affecting this route of entry into the plants [29].

AM fungi are known to be indirectly associated with bioremediation processes due to the so-called (mycor)rhizosphere effect which stimulates soil microbial activity, improves soil structure, and contributes to overall bioremediation of pollutants [36]. Other authors suggest that not only fungi but around the plant rhizosphere assists in phytoremediation process [37].

Furthermore, volatile or relatively volatile hydrophobic compounds such as HCH isomers, chlorpyrifos, diclofop-methyl, and trans-chlordane (Table 1) can be deposited on the aerial parts of plants [38].* Ricinus communis *L. showed high pesticide uptake values, which is consistent with the literature, since this process is enhanced by the lipid content of plant tissues and the surface area exposed to air [39].

The relationship between plants and organic pollutants in soil or the plant itself are involved. Plants could also be used to extract or degrade chlorinated organic compounds and other compounds, but the challenge lies in distinguishing between the direct action of the plants' metabolism and even their influence on microbial activity in soil.

The primary mechanism for dissipation of contaminants in the rhizosphere has been reported for PAHs [40], insecticides [41], and trichloroethylene [42]. The dynamics involved in plant-to-soil and plant-to-air transfer can be equated by bioconcentration ratios (BCRs) that correlate chemical concentrations in any vegetation tissues with concentrations in the soil in which the plant grows.

The BCR of all the pesticides under study was determined based on their ratio in* Ricinus communis* L. in the *T*_2_ experiment (Table 3). In the current scientific literature, the BCR is used to describe the ratio of the concentration of any molecule introduced into soil that supports vegetation to its measured concentration in plant parts. A plant-to-soil BCR expresses the ratio of a contaminant concentration relative to the mass of the same molecule in the soil [43].

The results of this study indicate that the higher the hydrophobicity of the organic compound and its molecular interaction with soil or root matrix the greater its tendency to concentrate in root tissues. Based on this finding, the pesticides under study were expected to show the following trend: HCHs < diclofop-methyl < chlorpyrifos < methoxychlor < heptachlor epoxide < endrin < o,p′-DDE < heptachlor < dieldrin < aldrin < o,p′-DDT < p,p′-DDT (increasing order of log *K*_ow_ values; Table 1). However, this is not entirely consistent with the root concentration pattern observed in the present study. The highest BCR values were found when* Ricinus communis *L. was used for the uptake of diclofop-methyl, methoxychlor, trans-chlordane, aldrin, p,p′-DDT, and o,p′-DDT.

These results suggest that despite predictions based on log *K*_ow_ be appropriate to model the transfer of some POPs, transfers occurs, in most often, without plants translocation, also due to the speed of displacement of ionic solution in the xylem.

There is a lack of empirical models to explain or predict the route of chemicals in air/plant/soil, where knowledge of the bioconcentration ratio (BCR) can be helpful in developing technologies for application in phytoremediation science [43].

Pesticide uptake is influenced by the chemical composition, especially in the present study, in which the composition of most of the target molecules gives nonpolar groups, and by the presence of lipids in the plant, listed in Table 3.

## 4. Conclusions

To our knowledge, this is the first report of a study of phytoremediation using* Ricinus communis* L. in soil contaminated with 15 POPs. The extent of bioaccumulation is found to depend on the physicochemical properties of each compound (mainly its hydrophobicity and volatility), on the plant species, and the type of tissue. The use of* Ricinus communis *L. may have some potential as a biotechnological approach for the decontamination of soils contaminated with organic pollutants, particularly because of its potential beneficial side effects of erosion control, site restoration, carbon sequestration, and feedstock for biofuel production. However, detailed and comprehensive studies on the remediation mechanisms of the selected plants are needed to test and validate effective rehabilitation methods in field conditions.

Future research will be required for in-depth studies of the biotic and abiotic mechanisms that may explain the decrease observed in the organochlorine concentration in the rhizosphere. The use of stable plants is an attractive method for decontaminating soils, especially in humid temperate climates, because of the small amount of handling they require and their low cost of maintenance.

## Figures and Tables

**Table 1:** Physicochemical properties of organochlorine pesticides.

Pesticide	Melting point (°C)	Density (g L^−1^, 20°C)	Vapour pressure (mm Hg, 20°C)	Solubility in water (*μ*g mL^−1^)	log⁡*K*_ow_^*∗*^
*α*-HCH	159-160	1.87	4.5 × 10^−5^	10	3.8
*β*-HCH	309-310	1.89	3.6 × 10^−7^	5	3.78
*γ*-HCH	112-113	1.85	4.2 × 10^−5^	7.3	3.61–3.72
Trans-chlordane	104-105	1.59–1.63	2.9 × 10^−5^	0.056	5.54
Chlorpyrifos	41-42	1.398 (43°C)	1.87 × 10^−5^	0.7	4.82
o,p′-DDE	88.4	No data	6.2 × 10^−6^	0.14	6
o,p′-DDT	74.2	0.98–0.99	1.1 × 10^−7^	0.085	6.79
p,p′-DDT	109	0.98–0.99	1.6 × 10^−7^	0.025	6.91
Diclofop-methyl	39–41	1.30 (40°C)	2.6 × 10^−7^	0.8	4.58
Aldrin	104–105.5	1.60 (20°C)	7.5 × 10^−5^	0.011	6.5
Dieldrin	176-177	1.75	3.1 × 10^−6^	0.110	6.2
Endrin	235	No data	2 × 10^−7^	0.2	5.6
Heptachlor	93	1.65–1.67	4 × 10^−4^	0.056	6.1
Heptachlor epoxide	160-161	1.91	1.95 × 10^−5^	0.0275	5.4
4,4′-Methoxychlor	89	1.4	Negligible	0.10	4.68–5.08

^*∗*^Distribution coefficients octanol-water (*K*_ow_).

**Table 2:** Limit of detection (LOD), limit of quantification (LOQ), mean recovery (level 0.1 *μ*g g^−1^), relative standard deviation (RSD), and calibration curve (*r* ^2^) for organochlorine pesticides in plant tissue (root, stem, and leaf) and soil samples.

OCPs	Plant tissue (root, stem, and leaf)				Soil			
	LOD (*μ*g L^−1^)	LOQ (*μ*g L^−1^)	Precision (RSD)	Recovery (%)	LOQ (*μ*g g^−1^)	Precision (RSD)	Recovery (%)	Calibration curve *r* ^2^
(1) *α*-HCH	0.002	0.007	8.5	95	0.008	8.8	105	0.9986
(2) *β*-HCH	0.001	0.008	6.7	82	0.007	6.4	99	0.9997
(3) *γ*-HCH	0.002	0.006	10.3	95	0.005	5.9	85	0.9982
(4) Chlorpyrifos	0.005	0.01	4.8	92	0.01	6.1	77	0.9995
(5) Heptachlor	0.001	0.005	7.2	81	0.008	7.2	75	0.9979
(6) Aldrin	0.001	0.007	9.1	88	0.01	3.9	91	0.9993
(7) Heptachlor epoxide	0.005	0.01	6.3	91	0.009	5.2	125	0.9981
(8) Dieldrin	0.002	0.006	4.9	82	0.01	6.0	93	0.9993
(9) Trans-chlordane	0.003	0.007	6.6	94	0.007	5.8	88	0.9978
(10) o,p′-DDE	0.002	0.005	7.1	90	0.008	7.3	97	0.9996
(11) Endrin	0.002	0.005	8.3	93	0.01	6.4	81	0.9988
(12) o,p′-DDT	0.002	0.005	3.9	83	0.01	5.9	97	0.9985
(13) p,p′-DDT	0.002	0.005	8.2	89	0.01	3.7	79	0.9982
(14) Diclofop-methyl	0.005	0.008	6.3	95	0.01	10.5	84	0.9991
(15) 4,4′-Methoxychlor	0.004	0.01	5.5	89	0.01	8.4	97	0.9975

**Table 3:** Root/shoot ratio and bioconcentration ratio (BCR) determined for the pesticide concentration in *Ricinus communis* L.

OCPs	*Ricinus communis* L.	
	Root/shoot (RSD)	Root/soil (RSD)
(1) *α*-HCH	0.014 (4.2)	0.773 (3.8)
(2) *β*-HCH	0.147 (4.4)	0.064 (6.1)
(3) *γ*-HCH	0.265 (6.1)	0.079 (6.8)
(4) Chlorpyrifos	0.453 (3.9)	0.123 (4.0)
(5) Heptachlor	0.565 (6.2)	0.875 (4.3)
(6) Aldrin	0.480 (5.7)	0.152 (6.2)
(7) Heptachlor epoxide	ND	ND
(8) Dieldrin	0.210 (5.2)	0.108 (5.8)
(9) Trans-chlordane	2.592 (4.1)	1.273 (4.5)
(10) o,p′-DDE	2.559 (6.0)	3.071 (5.5)
(11) Endrin	0.378 (6.1)	0.081 (7.0)
(12) o,p′-DDT	0.820 (6.2)	0.587 (6.5)
(13) p,p′-DDT	0.769 (5.8)	0.968 (4.2)
(14) Diclofop-methyl	3.668 (4.6)	2.085 (5.1)
(15) 4,4′-Methoxychlor	1.841 (3.8)	2.066 (3.5)
